# Ductal Carcinoma In Situ of the Breast: A Surgical Perspective

**DOI:** 10.1155/2012/761364

**Published:** 2012-09-04

**Authors:** Mohammed Badruddoja

**Affiliations:** Department of Surgical Oncology, Rehabilitation Associates of Northern Illinois, Rockford, IL 61111, USA

## Abstract

Ductal carcinoma in situ (DCIS) of the breast is a heterogeneous neoplasm with invasive potential. Risk factors include age, family history, hormone replacement therapy, genetic mutation, and patient lifestyle. The incidence of DCIS has increased due to more widespread use of screening and diagnostic mammography; almost 80% of cases are diagnosed with imaging with final diagnosis established by biopsy and histological examination. There are various classification systems used for DCIS, the most recent of which is based on the presence of intraepithelial neoplasia of the ductal epithelium (DIN). A number of molecular assays are now available that can identify high-risk patients as well as help establish the prognosis of patients with diagnosed DCIS. Current surgical treatment options include total mastectomy, simple lumpectomy in very low-risk patients, and lumpectomy with radiation. Adjuvant therapy is tailored based on the molecular profile of the neoplasm and can include aromatase inhibitors, anti-estrogen, anti-progesterone (or a combination of antiestrogen and antiprogesterone), and HER2 neu suppression therapy. Chemopreventive therapies are under investigation for DCIS, as are various molecular-targeted drugs. It is anticipated that new biologic agents, when combined with hormonal agents such as SERMs and aromatase inhibitors, may one day prevent all forms of breast cancer.

## 1. Introduction

Ductal carcinoma in situ (DCIS) of the breast is a noninvasive carcinoma with a wide spectrum of disease, ranging from low-grade to high-grade malignancy with foci of invasive malignancy. Histologically, DCIS is characterized by a proliferation of malignant cells in the ductal epithelium that are confined to the basement membrane and are not invading the normal breast parenchyma.

## 2. Epidemiology

Prior to advent of mammography, the diagnosis of DCIS was established only after excision of palpable lumps and histological examination of the tissue. Egan et al. [1], a radiologist based at the MD Anderson Cancer Center in Houston, Texas, is credited as the inventor of mammography in the late 1960s. By 1975, the widespread use of this imaging technique not only resulted in early detection of lesions in the breast but also led to a 60–70% reduction in morbidity and mortality from malignant diseases of breast [2]. The adoption of screening and diagnostic mammography resulted in an increase in the incidence of DCIS worldwide, with 80% of DCIS diagnosed by mammography. Currently, DCIS accounts for 20–25% of all newly diagnosed cases of breast cancer [3] and 17–34% of mammographically detected breast neoplasms [4, 5]. Approximately 1 of every 1300 screening mammograms results in a diagnosis of DCIS, and over 62,000 new cases of DCIS were diagnosed in 2009 [6].

Between 1983 and 2000 in the United States, there was a 500% increase in DCIS among women ≥50 years of age, though the incidence decreased by 2005 [7, 8]. Among women <50 years of age, DCIS incidence increased 290% from 1983 to 2003, followed by a continuous decline that was most likely due to a reduction in the use of hormone replacement therapy [9]. Virnig et al. [10] showed that the incidence of DCIS markedly increased from 5.8 per 100,000 women in the 1970s to 32.5 per 100,000 women in 2004, but then plateaued. DCIS is not common in younger women (<30 years of age). The risk of DCIS is 0.6 per 100,000 women 49–60 years of age, and increases to 1.4 per 100,000 women 70–84 years of age.

The risk of death from DCIS is very low; for women who were diagnosed between 1984 and 1989, the 10-year risk was 1.9% based on data from the National Cancer Institute (NCI) Surveillance, Epidemiology, and End Results (SEER) database [11]. The estimated incidence of DCIS was 32.5 per 100,000 women in 2004, based on NCI SEER data from 1975–2004. This is considerably higher than that reported in 1975 (5.8% per 100,000), but is consistent with the findings of the Swedish Two-County trial [12].The same trend is noted in numerous studies [13, 14]. In summary, there has been an overall increase in the incidence of DCIS in women after the age of 50 around the globe. This increase could be due to a greater awareness among women about breast malignancy, an increase in screening or diagnostic mammograms, the selective use of magnetic resonance imaging (MRI) in high-risk patients, or the use of genetic markers to identify high-risk patients, which will be discussed in detail below.

## 3. Risk Factors for DCIS

There are multiple risk factors for development of DCIS, including demographic, reproductive, biological, and behavioral risk factors. It is clear that the incidence of DCIS, like invasive carcinoma, is related to age. Incidence increases after the age of 50 years; several studies have shown that the incidence is 2.5 per 100,000 for women 30–40 years of age and steadily increases to a peak of 96.7 per 100,000 women 65–69 years of age. The incidence of DCIS is highest in Caucasian women compared with African American and Asians and Pacific Islanders, with the lowest incidence in Hispanics [15]. Prior to 1973, there were no data on the incidence of DCIS in urban and rural populations; however, one study showed that while the incidence of DCIS was increasing in both populations, the incidence was higher in urban women compared with rural women [16]. Another study showed that DCIS is also more prevalent in women who are less educated, particularly those with no high school degree [17]. One Australian study showed that the incidence of DCIS was 7.3% in those with relatively higher incomes compared to 4.5% in those with lower incomes.

Older age at menopause was associated with a higher incidence of DCIS [19]. One study showed that peri- and postmenopausal women had higher incidences of DCIS compared with premenopausal women [20]. The study, which was based on the Connecticut Tumor Registry, showed that there was a significant relationship between an increased risk of developing DCIS and older age at menopause; women who reached menopause after the age of 50 years had a higher risk of developing DCIS compared to those who reached menopause before the age of 45 years [20]. These findings are consistent with the likely role that hormone status plays in determining DCIS risk. A large prospective study from the United Kingdom reported a 56% increase in the risk of developing DCIS in women taking hormone replacement therapy (HRT), with the risk increasing with the duration of HRT [21]. Compared to those who never received HRT, women who took HRT for less than 5 years had significantly lower risk of DCIS. While the Iowa Women's Health Study found that there was no increased risk of DCIS in women who received HRT compared with those who did not [22], a subsequent metaanalysis found that women who had previously taken HRT had a higher risk of developing DCIS [22]. However, women who had used oral contraceptives (OCs) or were current users were found to have the same risk of DCIS as those who had never used OCs [23]. Nulliparity or women who had a late pregnancy (after 30 years of age) also had a higher incidence of DCIS [24]. Similar results were reported in a Danish cohort study [25]. Recently, the National Research Council of Australia published a summary of the evidence on HRT and the risk of breast cancer [26]. Only estrogen, combined estrogen-progesterone, combined estrogen-testosterone, and Tibolone are used for HRT. In women 50–79 years of age, the absolute risk of breast cancer is 38 per 100,000 for those taking combined estrogen and progestin (average over 5 years) compared with 30 per 100,000 for those who have never used combined HRT. It is not possible to accurately determine the duration of HRT after which breast cancer risk is increased; however, HRT for more than 3 years appears to be associated with an increased risk [27]. The Women's Health Initiative trial also showed that there is significant risk of developing breast cancer in women who had prior exposure to combined HRT for more than 6 years, while there is no overall increase in the incidence of breast cancer in women who were never exposed to HRT [28]. There are only inconsistent reports regarding the risk of breast cancer and the use of estrogen-only HRT. In one study, short-term use of estrogen-only HRT did not increase the risk of breast cancer [29]. In the Nurses Health Study, the risk of breast cancer was increased significantly in women with prior hysterectomy after 20 years or more of HRT, and the relative risk was higher for estrogen receptor (ER)+/progesterone receptor (PR)+ cancers [30]. One prospective study reported that combined use of estrogen and testosterone HRT in postmenopausal women increases the risk of breast cancer by 17% per year [31]. In the LIFT study of older postmenopausal women with low bone mineral density, breast cancer risk was decreased in the group receiving Tibolone compared with placebo; however, the study was terminated early due to an increased incidence of stroke [32]. There does not appear to be a significant difference in the risk of breast cancer based on the route of HRT administration (e.g., transdermal, oral, implant [33]).

A history of benign biopsied breast disease is associated with a higher risk of breast cancer [34]. In addition, the same study indicated that high intake of vitamin A and alcohol increases the risk of breast cancer. Obesity and increases in BMI by 25% are also risk factors for breast cancer. In a study conducted in Alberta, Canada, by Friedenreich and colleagues, increased physical activity decreased the serum levels of all sex hormones [35], previously sedentary postmenopausal women who adhered to a moderate-to-vigorous intensity exercise program showed decreases in serum levels of estrogen, progesterone, testosterone, and sex hormone binding globulin and had a lower risk of postmenopausal breast cancer. In addition, the exercise program led to weight loss which also contributed to the decrease in breast cancer risk. 

Mammographic detection of increased density of breast tissue is also a risk factor for breast cancer. In a recent study by Boyd and colleagues, women with 75% or higher density on a mammogram had an increased risk of breast cancer compared with women with mammograms with less than 10% density [36]. Women with extensive mammographic density detected between screening tests are also at high risk of developing DCIS, and considerable number of DCIS cases are associated with this single risk factor. Thus, high-risk patients who have dense breast tissue detected by mammogram should have a follow-up MRI of the breast so that lesions are not missed.

Observational studies have suggested that beta carotene, vegetables, fruits, and antioxidants may have protective effects against breast cancer. However, a recent randomized controlled trial found no such protective effects of a diet supplemented with beta carotene, vitamin C and E, fruits, and other antioxidants [37]. This trial followed 624 women for a period of 9.4 years and found that compared to placebo group, the relative risks of developing breast cancer were 1.11% in vitamin C group, 0.79% in vitamin E group, and 1% in beta carotene group. The Iowa Study showed that aspirin and nonsteroidal anti-inflammatory drugs (NSAIDs) have a protective effect against breast cancer [38]. An animal and in vitro study also showed that aspirin not only prevents breast cancer, but also prevents metastasis in breast cancer [39].This was a prospective observational study in which 4164 female patients with stage I, II, or III breast carcinoma were followed for 26 years [39]. The relative adjusted risk of metastasis in those who took aspirin for 1 day, 2 to 5 days, and 6 to 7 days per week compared with those who did not take aspirin were 1.07 (95% CI, 0.70 to 1.63), 0.29 (95% CI, 0.16 to 0.52), and 0.36 (95% CI, 0.24 to 0.54), respectively. The most recent study, based on a systematic comparison of evidence from observational studies, indicated that daily aspirin intake not only prevents colorectal cancer, but also prevents breast, esophageal, biliary, and gastric cancer [40]. The study also showed that aspirin prevents and delays metastasis from breast, esophageal, gastric, and hepatobiliary cancer. 

## 4. Diagnosis

Diagnosis of DCIS is primarily based on imaging results in developed countries of the world, while developing and or underdeveloped countries of the world continue to rely on excision and histologic analysis of the tissue biopsy. This disparity is due to the lack of screening facilities and imaging equipment, lack of funding, and cultural barriers in certain developing and underdeveloped countries [41]. In these countries, only 20% of ductal carcinomas are diagnosed on clinical examination and 80% of patients present to the clinician with a palpable lump, nipple discharge, or skin change over the breast [42]. Very recently, the US Cancer Prevention Task Force made a very cautionary remark regarding the overuse of imaging studies (i.e., mammography) for evaluation of diseases of breast [43]; however, even with such cautionary remarks, mammography remains the gold standard for diagnosis of breast diseases. Clinicians continue to advocate for yearly screening mammography in low-risk or non-risk patients after the age of 50 years and in high-risk patients before age of 40 years. Nevertheless, there is concern about radiation exposure during screening mammography. Advances in digital mammography have led to a 22% reduction in radiation exposure compared with film mammography [44]. While digital mammography is more expensive than film mammography, some claim that digital mammography is able to detect more lesions. In one study [45], the detection rate of each modality is same, but the sensitivity and specificity of digital versus film mammography vary with age, tumor characteristics, breast density, and menopausal status. The detection rate was higher with digital mammography than film mammography in women between the ages of 60 and 69 years (89.5% versus. 83%; *P* = 0.014) and in those with ER+ cancers. Clinicians and radiologists will have to decide which imaging study is appropriate for an individual patient. 

MRI is used frequently after the detection of lesions by digital or screening mammography, primarily because MRI can help guide surgical decision making among the possible options—breast conserving surgery, mastectomy, or bilateral mastectomy. Surgical decision making is based on the multicentricity of the disease, tumor size, status of the contralateral breast, and family history. The accuracy of MRI over mammography in evaluating the first 3 factors defines its clinical value. In addition, MRI has a higher diagnostic accuracy for DCIS compared with either film or digital mammography. Riedl and colleagues [46] studied 672 imaging rounds in a high-risk population and found that the detection rate of DCIS by mammography, ultrasound (US), and MRI were 50%, 42.9%, and 85.7%, respectively. This detection rate is similarly high for both in invasive cancer and preinvasive cancer (DCIS). Based on their findings, the authors recommended that MRI should be included in the imaging of high-risk patients. Kuhl and colleagues [47] performed a retrospective study and found that the detection rate of DCIS is 92% by MRI and 56% by mammography, and that 48% of high-grade DCIS are missed on mammography but detected by MRI. 

Collectively, the literature reports that MRI is able to detect multicentric lesions, estimate the size of the tumor, and predict the invasive nature of the lesion. Hwang and colleagues [48] found that MRI has 94% sensitivity in detecting multicentric lesions compared to mammography, which has 38% sensitivity. In a similar study, Menell and colleagues [49] found that the sensitivity of MRI and mammography for detecting multicentric lesions in DCIS are 94% and 38%, respectively. However, in another study of 86 women, Santamaría and colleagues [50] did not find any difference in sensitivity between MRI and mammography for detecting multicentric lesions in DCIS, but did report a higher performance of MRI than mammography. Hollingsworth and Stough [51] reported that the incidence of occult multicentric disease in DCIS was 6.3% and could be detected by MRI. Multicentricity is defined as a lesion 5.0 cm from the index lesion or discontinuous growth into another quadrant of the breast [52]. Assessment of the growth patterns of DCIS during tissue processing and histologically based tumor measurements are difficult, as the three-dimensional (3-D) extent of the disease must be reconstructed using 2-D pathology slides. For this reason, MRI measurements of tumor size may be more accurate than pathologic measurements. Uematsu and colleagues [53] found that MRI measurement of DCIS is more accurate than mammographic measurement of the extent of disease, and Lehman and colleagues [54] reported that the sensitivity rate of MRI in detecting contralateral DCIS is 77%. Interestingly, MRI results led to biopsy of the contralateral breast in 18 patients, of which only 28% were positive.

Ultrasound (US) also plays a significant role in diagnosis of DCIS. Gwak et al. [55] conducted a retrospective study of US for detecting DCIS and found that US is more accurate than mammography. In a similar study by Moon et al. [56], DCIS detected by US that has speculated margins, marked hypoechogenicity, thick echogenic margin, and posterior acoustic shadowing is highly suggestive of invasion.

The literature shows that each and every imaging technique available for detecting DCIS has pitfalls and limitations, but each technique also complements one other. Clinicians and radiologists will need to decide which imaging technique is appropriate for their particular patient, taking into consideration her age, family history, and other risk factors. It must be pointed out, however, that MRI is very expensive; in the United States, the average cost of a breast MRI is $5000.00 [57]. For this reason, the indications for preoperative MRI for the diagnosis of breast diseases are clearly stated in the literature and guidelines and ensure that only selected patients undergo breast MRI [58]. 

Complete physical examination is mandatory after discovery of a possible lesion or lesions on an imaging study; however, the ultimate diagnosis of the lesion still depends on the histology of a biopsy specimen. Currently, the 3 most popular methods of biopsy are needle biopsy, core needle biopsy (CNB), and vacuum-assisted biopsy. Fine-needle aspiration (FNA) is not adequate to establish a diagnosis of DCIS [59]. With advances in imaging, CNB can now be performed as a US-guided or stereotactic procedure by the radiologist. CNB is the best method for establishing the histologic diagnosis of DCIS. Vacuum-assisted biopsy has a high specificity and sensitivity, but may still miss a diagnosis of DCIS in 17% of cases [60]. 

Prior to image-guided biopsy, needle localization excisional biopsy was a very common practice for treating DCIS. This technique has the advantages of completion of therapy, provided a negative margin is obtained, and is therefore highly cost effective [61]. However, this method is rarely used today for treating DCIS. 

Preoperative variables of CNB for diagnosis of DCIS are significantly associated with under-staging of the disease and include experience of the operator, biopsy device, guidance method, size, mammographic features, and palpability of the neoplasm [62]. Based on these variables, 25% of DCIS may have invasive carcinoma and the treatment plan may change. The best approach is to take multiple samples during CNB in order to establish an appropriate histologic diagnosis. CNB specimens must have adequate tissue so that the pathologist can determine the prognostic factors, degree of invasiveness based on nuclear grading, presence or absence of necrosis, mitotic figure, hormonal status of the lesion, and the presence of molecular markers.

## 5. Pathologic Classification

DCIS is not a single entity, but rather a spectrum of disease; in essence, it refers to malignant change in the ductal epithelium. Because of the heterogenicity of DCIS lesions, no single satisfactory pathological classification system has been adopted. The traditional classification system is based on morphology, architecture, and nuclear grading of the lesion [63], as well as on the presence or absence of necrosis. Silverstein et al. [64] proposed the division of DCIS into 3 groups: high grade, non-high grade with comedonecrosis, and non-high grade without comedonecrosis. One study [65] reported that 23 pathologists were in complete agreement using 5 different classification systems, including that proposed by Silverstein. The International Consensus Conference has failed to endorse any classification, but recommended that pathology reports must include information on nuclear grading, necrosis, polarization, and architectural pattern [66]. Allred [67] proposed the following clarification.

- Comedo group—large cell: more aggressive form also referred to as comedocarcinoma.
- Noncomedo group—small cell: less aggressive and is further divided into:
  - cribriform,
  - micropapillary,
  - solid.
- DCIS can also be classified depending on the presence of central necrosis [68]:
  1. DCIS with no central necrosis (noncomedo group).
  2. Low grade with no central necrosis:
    1. low grade DCIS,
    2. intermediate grade DCIS: well differentiated, cribriform, micropapillary, solid- and small-cell type.
  3. DCIS with central necrosis:
    1. poorly differentiated,
    2. comedo type,
    3. large-cell type.

Such classifications may be clinically meaningful in terms of describing the invasive potential of the neoplasm and informing the treatment plan to avoid overtreatment. In the past, nuclear grading using microscopy could not predict which DCIS would develop into invasive carcinoma. Chapman and colleagues [69] found that nuclear grading by image analysis does have prognostic value, with quantitative nuclear image features able to predict which DCIS will transform into invasive cancer. While the traditional classification is probably going to be changed based on hyperplasia of the duct or epithelium, because an element of subjectivity in the microscopic interpretation of these hyperplastic lesions persists, it is very unlikely that this issue will be resolved soon. A new classification system was proposed based on the presence of mammary intraepithelial neoplasia (MIN) of epithelium either in the duct or lobule, followed by grading in accordance with the trend in many other sites [70]. Most recently, Tavassoli [18] proposed a completely new classification system with some modifications as proposed by Rosai [70]. These classification systems are based on the presence of ductal intraepithelial neoplasia (DIN; Table 1).

Such new classifications have certain advantages and obvious merits, and it is possible that a modified version of the DIN classification will eventually be adopted, though not in the near future. Recently, Costa and Zanini [71] have questioned whether DIN is really a malignant lesion. Guerrieri-Gonzaga and colleagues [72] published their experience in treating 1267 cases of DIN and showed that it is a potentially malignant lesion and should be treated either by BCS or by chemopreventive therapy. These authors have accepted the new pathological classification.

Recently, considerable efforts have been made in evaluating potential molecular markers of DCIS. However, most studies have shown that candidate molecular markers of DCIS have little prognostic value [73, 74]. Approximately 70% of DCIS express ER [75], which is normally expressed by luminal epithelium. Almost 50% of DCIS express HER2/neu [76]. Mutated p53 (a tumor suppressor gene) is expressed by about 25% of DCIS [77]. DCIS of solid, flat or micropapillary type exists in the basal phenotype of breast cancer and demonstrates the same immunophenotype as invasive breast cancer [78]. Androgen receptor (AR) has been detected in female breast cancer and is often associated with apocrine differentiation. Inherited differences in AR CAG length might influence the transition from DCIS to invasive carcinoma, perhaps by modulating the function of AR in breast tissue [79]. All DCIS express E-cadherin, but lobular carcinoma shows focal loss of E-cadherin or complete lack of membrane staining. It is sometimes difficult to differentiate histologically between lobular carcinoma and ductal carcinoma, and E-cadherin immunohistochemical studies can be used to differentiate between the 2 groups of in situ carcinoma of breast [80]. In the future, molecular markers may help to predict which group of DCIS will become invasive carcinoma. 

New technologies, such as array-based CGH, RNA expression profiling, have proven to be of great value in distinguishing between poorly differentiated and well-differentiated DCIS by detecting quantitative difference in gene expression. Proteomic analysis also may be able to predict which DCIS will become invasive carcinoma and will help inform the appropriate treatment [81]. Kerlikowske and colleagues [82] conducted standardized pathology reviews and immunohistochemistry staining for ER, PR, Ki67 (tumor proliferating index) antigen, p53, p16, epidermal growth factor receptor-2 (ERBB2, HER2 neu oncoprotein), and cyclooxygenase-2 (COX-2) in paraffin-embedded DCIS tissue. They found that DCIS lesions positive for p16, COX-2, and Ki67, or those detected by palpation are more likely to develop into invasive cancer. Radisky and colleagues [83] examined the significance of p16 INK4a expression in women with atypical hyperplasia and found that expression was not a risk for breast cancer. Most recently, a study by Adler and colleagues [84] found that the vascular pattern is not a predictor of aggressive behavior of DCIS, suggesting that DCIS biology is independent of angiogenesis. We do not yet know whether this also means that vascular endothelial growth factor (VEGF) does not control the aggressive nature of DCIS.

## 6. Treatment Plan

The goal of DCIS treatment is complete removal of the neoplasm, if possible, and prevention of recurrence. Local treatment of DCIS is simple mastectomy, lumpectomy (though there is usually no lump when DCIS is diagnosed by imaging), lumpectomy with post lumpectomy radiation, quadrantectomy without or with postquadrantectomy adjuvant treatment, followed by chemopreventive therapy. Traditionally, simple mastectomy is the curative treatment for 98% of cases of DCIS, and the local recurrence rate is very low [85]. However, even after simple mastectomy there may be local recurrence. The causes of such local recurrence are either a missed diagnosis of invasive carcinoma during the original surgery or incomplete removal of breast tissue, especially from the skin flap in nipple-sparing mastectomy. Such recurrence occurs only in 1% to 2% of cases [86].

This traditional treatment of DCIS has been challenged by Fisher and colleagues [87] who conducted a randomized trial that demonstrated that total mastectomy and breast conservative surgery for DCIS are associated with equivalent outcomes. Nevertheless, there are certain indications for complete mastectomy for DCIS, beyond the preference of the patient and/or the physician. The indications for total mastectomy as determined by a joint committee of the American College of Surgeons, American College of Radiology, and the American College of Pathologists are women with 2 lesions in the same breast; diffuse malignant appearing lesion in the breast; persistent positive margin after lumpectomy and cavity shaving with multiple attempts; inability to give radiation due to prior radiation or presence of SLE; radiation treatment is not available, especially in underdeveloped countries; extensive DCIS where the tumor is removed with a very small negative margin; tumor size and breast size will produce poor cosmetic result; pregnancy. 

Currently, lumpectomy with no radiation in the low-risk patient or with radiation following surgery is the standard of care in the United States and other developed countries [88]. However, this standard of care is not possible in underdeveloped countries where DCIS is detected by the patient or by the physician as either palpable lump, discharge from nipple, or skin dimpling, rather than by screening or diagnostic imaging studies due to lack of resources [41]. In addition, there are also limited capabilities for radiation treatment following local excision of resectable tumors in developing and underdeveloped countries. Therefore, in these areas, the standard of care is simple mastectomy for all stages of DCIS. 

Studies have shown that there is a low recurrence rate of DCIS with excision alone as compared to excision and radiation, especially in low-risk patients [89, 90]. For this reason, it is worthwhile to further explore the possibility of breast conserving surgery alone, especially in patients with low risk. Three randomized trials have compared the outcomes with excision alone versus excision and radiotherapy [91–93]. These trials showed that addition of radiation therapy significantly reduces the risk of recurrence by 40% in the ipsilateral breast. Multiple observational studies, though less powerful than the NSABP-17 trial, also showed lower rates of local recurrence of DCIS or invasive cancer for women undergoing breast conserving surgery followed by radiation, although not all reported statistically significant differences [94–96]. Observational studies from Sweden indicate no mortality benefit associated with breast conserving surgery with radiation compared to breast conserving surgery alone [97]; these results were echoed in one other study [98]. Though these results are from observational studies, taken together, there is no evidence that conservative surgery plus radiation is more or less effective than breast conserving surgery alone. This lack of differential effect can be seen across all of the most important prognostic factors, including grade, tumor size, involved margins, and comedonecrosis.

It is felt that an involved margin is one of the most important prognostic factors for recurrence, yet it is still not agreed what should be the safe margin of lumpectomy [99]. In their review, Revesz and Khan do not provide any specific safe margin; rather, they state that until better data are available, the desirable margin will vary depending on individual factors, including age, histology, and patient preference. It is likely that a safe margin is “ink should not touch the margin of the excised mass and should be at least 2 mm from the surgical margin,” as stated by Ruggiero et al. in [42]. The margin varies in the literature, from 1 mm to 3 mm [42]. Blair et al. [100] reported that in the United States, only 48% of surgeons perform cavity and bed shaving, very few undertake frozen section analysis or imprint cytology, and 57% never reexcise with positive margins. The literature suggests that there is still controversy as to whether all patients should be treated with radiation after lumpectomy. Jiveliouk and colleagues [101] recently reported their experiences with the treatment of pure DCIS using lumpectomy and postoperative external beam radiation in an Israeli population; during an 8-year follow-up period, the overall survival, disease-free survival, and event-free survival were 100%, 100%, and 87%, respectively. This is a unique result and may be due to early initiation of treatment or that the biological behavior of DCIS in Israeli women is different from that in women from other Western countries. Kayani and Bhurgri [102] also reviewed their experience with 38 women with DCIS in Karachi, Pakistan; they found that, if untreated, only 40% of cases were aggressive and 60% were very indolent. Most likely, the biological behavior of DCIS is different in Pakistani women such that they do not develop the more aggressive types of DCIS. The Eastern Cooperative Oncology Group recently reviewed their experiences with local excision without radiation for DCIS [103]. Patients with either low or intermediate risk, with tumors measuring 2.5 cm or smaller or high-grade or DCIS 1 cm or smaller who had microscopic margins of 3 mm or wider, were eligible for study. During the 6.2 years of followup, the 5-year rate of cancer recurrence and related morbidity was 6.1% in patients with low or intermediaterisk, and 15.3% in the high-grade DCIS group. This study proves that all patients with DCIS do not need radiation after lumpectomy. Very recently, similar study has been carried out by Ruggiero et al. [42] in 161 patients who were followed for 5 years; the recurrence rate was 6.2% in the group of patients who had only quadrantectomy without radiation therapy. According to these authors, the risk factors for local recurrence were age <45 years, positive margin <2 mm, and grade 3 neoplasm. Based on their findings, the authors recommended adjuvant radiotherapy in patients who had these risk factors for local recurrence. 

Taken together, the current trend is that only high-risk patient should undergo adjuvant radiotherapy after lumpectomy. Typically, 50 Gy of external beam radiation is administered in 25 fractions. There is also growing interest in balloon brachytherapy for treatment of DCIS following lumpectomy, which would allow for accelerated breast radiation therapy. The literature contains reports of satisfactory results with balloon brachytherapy in DCIS in terms of disease-free survival and cosmoses [104, 105]. However, a recent report about the long-term result of brachytherapy for treatment of DCIS was presented at the San Antonio Breast Cancer Symposium (2011) and published by the American Association for Cancer Research [106]. In this study, Smith et al. at MD Anderson Cancer Center reviewed the medical records of 130,535 patients who underwent brachytherapy for DCIS and then were followed for 5 years. Surprisingly, 50% of the patients eventually underwent complete mastectomy, either due to complications of the brachytherapy or recurrence of the tumor. Multiple studies have demonstrated that MRI detects multiple foci in 10%–30% of patients with DCIS, and that neither mammography nor US can detect these metacentric lesions [107]. When these patients are treated with balloon brachytherapy, they are inadequately treated; this may explain why there is such a high recurrence rate of DCIS after brachytherapy. It is therefore appropriate that patients undergo MRI evaluation prior to brachytherapy, and if multiple foci are present, then either these patients should undergo total mastectomy or total breast radiation.

Controversy exists regarding the treatment of micrometastasis in DCIS. Micrometastasis should be treated either with axillary dissection, chemotherapy, or radiotherapy [108]. DCIS is a part of breast and ovarian cancer syndrome [109], and the BRCA1 and BRCA2 mutation rate in invasive carcinoma is same as that in DCIS. These findings suggest that a patient with personal and family history of breast cancer and or ovarian cancer should be followed very closely as per the risk protocol for breast cancer.

The value of genetic testing for BRCA1 and BRCA 2 mutation is its ability to reduce the number of women who develop breast cancer and the number of women who die of disease. Patients with BRCA1 and BRCA2 mutations have several options for breast cancer prevention. These options include prophylactic total mastectomy, prophylactic bilateral oophorectomy and chemoprevention with SERM, third generation aromatase inhibitors, or raloxifene. Statistical analyses indicate that total mastectomy reduces the risk of developing breast cancer by 89% [110]. Though prophylactic total mastectomy offers the best protection against developing breast cancer in BRCA1 and BRCA 2 mutation carriers, one study in Canada showed that the majority of women with BRCA1/2 mutations are unwilling to undergo such a radical surgical procedure [111].

The NSABP-24 trial assessed the value of tamoxifen following the diagnosis of DCIS and found that treatment reduces the recurrence rate of DCIS or invasive carcinoma in the ipsilateral breast. The effect of tamoxifen is highly significant for patients with ER+ DCIS, whereas the effect in reducing recurrence DCIS in the ipsilateral breast is not significant in ER− DCIS [112]. The same trial also found that tamoxifen therapy was associated with a 50% reduction of DCIS or invasive carcinoma in the contralateral breast, but had no impact on all-cause mortality. Combined treatment (lumpectomy, radiation, and tamoxifen) compared with lumpectomy and tamoxifen reduced the overall rate of cancer 29% [113]. This study also showed that tamoxifen is less effective in patients without comedonecrosis or who have smaller tumors. The unwanted effects of tamoxifen include hot flashes, fluid retention, vaginal discharge, osteoporosis, thromboembolic disease, and endometrial carcinoma. A study by Cuzick and colleagues [114] reported that the risk reduction of breast cancer with tamoxifen persists for at least 10 years, but that most side effects do not continue after 5 years. However, an observational study by Warren and colleagues [115] found that women with DCIS who receive tamoxifen had the same hazard of local recurrence of DCIS or invasive cancer as women who did not receive tamoxifen. 

In addition to tamoxifen, other SERMs such as raloxifene and lasofoxifene are also used as chemoprevention agents. The STAR trial, MORE trial, and CORE trial have studied the role of raloxifene for prevention of breast cancer and have shown positive results [116]. However, a recent study by Viring and colleagues [117] reported that while raloxifene reduced the risk of invasive breast cancer, it was not associated with decreased incidence of DCIS. 

Currently, third-generation aromatase inhibitors (anastrozole, letrozole, and exemestane) are also used as chemoprevention agents with greater specificity and fewer side effects [118]. However, all of these chemopreventive drugs have no impact on ER-tumors and this remains a challenging area for breast cancer prevention. Possible agents for prevention of ER-neoplasms include cyclooxygenase-2 inhibitors, statins, and vitamin D analogs. Yet none of these drugs has been tested in humans in a randomized controlled trial, which is necessary to prove the efficacy of these drugs for prevention of breast cancer. There is one laboratory study in progress that is evaluating inhibition of p38 kinase as a chemopreventive measure for ER-breast tumors [119]. The p38 kinase causes cell proliferation, and most ER-negative breast neoplasms overexpress p38 kinase. This preliminary study will provide the foundation for new approaches to the treatment or prevention of ER-breast neoplasms.

Rexinoid LG100268 [120] has been shown in animal models to be an effective chemopreventive agent for prevention of preinvasive neoplasm of the breast with minimal toxicity [121]. Future trials are needed in humans to assess the clinical translation of this chemopreventive agent. PPAR-*α* and PPAR-*γ* ligands induce apoptotic and antiproliferative responses, respectively, in human breast cancer cells, and their activation is associated with specific changes in gene expression [122]. Therefore, PPAR-selective retinoids may also be potential chemopreventive agents.

The issue of sentinel lymph node (SLN) biopsy in DCS has been extensively studied by various investigators. Tada and colleagues [123] have found that the incidence of positive SLN is 1.25% and 6.8% in DCIS and intraductal carcinoma (IDC), respectively. Intra et al. [124] studied the incidence of positive SLN in 854 patients with DCIS. They found the incidence of positive SLN was 4%, or 12 cases. Of these 12 cases, 7 had micrometastases with tumor size <2 mm and 5 had macrometastases with tumor size >2 mm. Four additional cases had isolated tumor cells (ITC). Julian and colleagues [125] reviewed the records of 813 patients with DCIS. These patients were studied under the auspices of the NSABP B-17 and B-24 projects. The NSABP B-17 investigators found that 7 patients developed ipsilateral nodal recurrence (INR) and the overall INR rate was 0.83 per 1000 patient-years. In NSABP B-24, the overall INR rate was 0.36 per 1000 patient-years. It was concluded that INR can be considered a surrogate for axillary involvement at the time of diagnosis of DCIS. These findings suggest that the rate of positive SLN in DCIS is so low that there is generally no indication for performing SLN biopsies in patients with DCIS. However, if at any time the patient undergoes complete mastectomy, then the patient must also have an SLN biopsy. Other relative indications of SLN biopsy are perineural invasion and high grade with comedonecrosis.

Various treatment options should be discussed with the patient with DCIS. Patients should be told in detail about the biological behavior of the DCIS, with special reference to the natural history of the disease, the outcomes of various types of treatment, the recurrence rate after treatment, the results of salvage treatment in the event of recurrence, the risks and benefits of the various treatment options, and the disease-free survival and overall survival rates of the various treatments. The patient and possibly family members should be actively involved in treatment decision making. Katz and colleagues [126, 127] conducted a population-based cohort study of 659 women from the Detroit and Los Angeles areas who were diagnosed with DCIS in 2002 to examine the role of the patient in treatment decision making and how the patient's input affected the treatment. In the study, greater patient involvement in the decision-making process led to larger number of mastectomies. Furthermore, the Katz study showed that only 13.1% of women were not influenced by their physicians concerns about recurrence and underwent mastectomy compared to 48.5% who were greatly influenced by the possibility of recurrence. This finding suggests that the engagement of a knowledgeable surgeon in the treatment discussion can be a very powerful tool in guiding the treatment of a particular patient. The surgeon must consider all the risk factors of recurrence after the definitive curative treatment of DCIS and must work with the oncologist to select adjuvant treatment, if needed, and chemopreventive treatment. 

## 7. Conclusion

DCIS is a heterogeneous neoplasm whose biological behavior is still incompletely understood. Epidemiologic studies show that with the advent of various imaging techniques, the incidence of DCIS increased and has reached a plateau in past decade. Approximately 80% of DCIS cases are diagnosed by imaging studies in developing countries, whereas the majority of cases in developing or underdeveloped countries present as palpable lumps, nipple discharge, and comedonecrosis. Each of the imaging modalities has advantages and limitation, but they can complement each other to achieve an accurate diagnosis of DCIS. Definite histological diagnosis of nonpalpable DCIS is established by either needle- or US-guided CNB or stereotactic biopsy or vacuum-assisted biopsy. Currently, CNB is best technique to obtain an accurate histologic diagnosis. Various treatment options are available. The gold standard of treatment of DCIS in developed countries is wide local excision of the tumor with negative margins followed by external beam radiation. Such treatment options may not be available in underdeveloped countries, where total mastectomy is the treatment of choice. Surgery and radiation are superior to surgery alone with regard to recurrence, but there is no benefit in terms of overall survival for either of these approaches. Long-term survival is possible, even in underdeveloped countries without treatment. Additional research is needed to determine the role of balloon brachytherapy as an adjuvant treatment. Tamoxifen is a beneficial adjuvant therapy. Further research to understand the biological nature DCIS will resolve some of the remaining controversy about the best treatment for DCIS. Various preventive measures are available to protect against the development or progression of DCIS, including surgical and nonsurgical interventions. Investigations are ongoing regarding molecularly targeted drug development for prevention and treatment of DCIS.

## Figures and Tables

**Table 1:** Classification of Tavassoli [18].

Proposed classification	Current designation	Necrosis	Excised margin
DIN 1a	IDH	None	Negative

	AIDH, flat monomorphic	−	? Negative
	DCIS, grade 1 (crib/micropap)	−	Positive
DIN 1b	DCIS, grade 2	+	Positive
	(crib/micropap + necrosis or atypia)	+	
	Special type—specify		

DIN 3b	DCIS Grade 3	+ + +	Positive
	(Anaplastic DCIS)	±	
